# Relationship between quadriceps thickness and 60-second sit-to-stand test in patients with chronic kidney disease

**DOI:** 10.1590/2175-8239-JBN-2021-0064

**Published:** 2021-09-01

**Authors:** Marthley J. C. Costa, Frederico C. B. Cavalcanti, Shirley Dias Bezerra, José Candido de Araújo, Juliana Fernandes, Patrícia E. M. Marinho

**Affiliations:** 1 Universidade Federal de Pernambuco, Departamento de Fisioterapia, Programa de Pós-Gradução em Fisioterapia, Recife, PE, Brasil.; 2 Real Hospital Português de Beneficência em Pernambuco, Unidade de Nefrologia, Recife, PE, Brasil.; 3 Universidade Federal de Pernambuco, Departamento de Fisioterapia, Laboratório de Fisioterapia e Saúde Pública, Recife, PE, Brasil.

**Keywords:** Musculoskeletal System, Ultrasonics, Body Composition, Physical Functional Performance, Sistema Musculoesquelético, Ultrassom, Composição Corporal, Desempenho Físico Funcional

## Abstract

**Background:**

This study aimed to evaluate the relationship between quadriceps muscle thickness and functional performance on the 60s sit-to-stand test (60s-STS), the six-minute walk test (6MWT), and handgrip strength in non-dialytic stage 4 and 5 chronic kidney disease (CKD) patients.

**Methods:**

This was a cross-sectional study that evaluated 40 CKD patients aged between 30-70 years. Participants were submitted to an assessment that included quadriceps muscle thickness evaluated by a portable ultrasound. Functional performance tests included the 60s-STS, distance walked in the 6MWT, and handgrip strength. Also, body composition evaluated using electrical bioimpedance analysis and physical activity level through the short version of International Physical Activity were measured. Multiple linear regression was used to investigate the relationship between the quadriceps thickness and functional performance.

**Results:**

Quadriceps muscle thickness was correlated to 60s-STS (R2 = 43.6%; 95% CI = 0.022 - 0.665; β = 0.34; p = 0.037). Also, a moderate correlation between this muscle thickness and appendicular skeletal muscle (ALM) was found in CKD patients (r = 0.603, p <0.001). No relationship was found between quadriceps muscle thickness with the 6MWT and handgrip strength.

**Conclusion:**

Quadriceps muscle thickness is associated to 60s-STS, thus our results demonstrate the repercussions of the disease on the musculoskeletal system.

## Resumo

**Antecedentes:**

Este estudo teve como objetivo avaliar a relação entre espessura muscular do quadríceps e desempenho funcional no teste *sit-to-stand* de 60s (STS-60s), no teste de caminhada de seis minutos (TC6M), e na força de preensão manual em pacientes não dialíticos com doença renal crônica (DRC) estágios 4 e 5.

**Métodos:**

Este foi um estudo transversal que avaliou 40 pacientes com DRC com idades entre 30-70 anos. Os participantes foram submetidos a uma avaliação que incluiu a espessura muscular do quadríceps avaliada por um ultrassom portátil. Os testes de desempenho funcional incluíram o STS-60s, a distância percorrida no TC6M e a força de preensão manual. Além disso, foram medidos a composição corporal, avaliada usando a análise de bioimpedância elétrica e o nível de atividade física por meio da versão curta do International Physical Activity. A regressão linear múltipla foi usada para investigar a relação entre a espessura do quadríceps e o desempenho funcional.

**Resultados:**

A espessura muscular do quadríceps foi correlacionada com o STS-60s (R2 = 43,6%; IC 95% = 0,022 - 0,665; β = 0,34; p = 0,037). Também foi encontrada uma correlação moderada entre esta espessura muscular e a massa muscular esquelética apendicular (MEA) em pacientes com DRC. (r = 0,603, p <0,001). Nenhuma relação foi encontrada entre a espessura muscular do quadríceps com o TC6M e a força de preensão manual.

**Conclusão:**

A espessura muscular do quadríceps está associada ao STS-60s, assim nossos resultados demonstram as repercussões da doença sobre o sistema músculo-esquelético.

## Introduction

Dysfunction in the musculoskeletal system is frequent in chronic kidney disease (CKD) as a result of systemic changes caused by decreased glomerular filtration rate (GFR), inflammatory process, metabolic acidosis, reduced protein intake, insulin resistance, and physical inactivity^1^^-^ 5. This condition contributes to the development of uremic sarcopenia, characterized by reduced strength and muscle mass^5^^,^ 6. Previous studies have shown that the prevalence of sarcopenia in non-dialytic (non-D) CKD patients may range from 5 to 60%^4^^,^ 7 ^,^ 8, and is associated with physical limitations, reduced quality of life, and increased morbidity and mortality^4^^,^ 8.

The reduction in muscle mass in patients with CKD becomes apparent in the beginning of the course of this disease and it is multifactorial, since the disease itself contributes to the catabolic state due to increased muscle proteolysis and reduced protein synthesis^5^^,^ 9. This reduction in muscle mass leads to sedentary lifestyle, exercise intolerance, and poor cardiorespiratory fitness^5^^,^ 9 ^-^ 11 being in turn associated with functional limitation and increased mortality^9^^,^ 12.

Magnetic nuclear resonance (MRI) and computed tomography (CT) have been considered the gold standard for evaluating muscle mass quantification. However, lack of portability, high cost, and radiation exposure limit its use in clinical practice^6^^,^ 12 ^,^ 13. In this way, ultrasonography (US) has been proposed as an alternative means for evaluation because it is valid, reliable, and a low cost imaging method with good intra and inter-evaluator reliability, and is feasible in clinical practice^12^^,^ 13.

Considering the existence of muscle mass loss during CKD and its clinical repercussions on inactivity and functionality, assessing quadriceps muscle thickness in relation to the functional performance of these patients may provide information on the course of muscle mass loss and functional decline throughout kidney disease. Previous studies have demonstrated positive correlation and good reliability and validity among US with DEXA, CT, and RMI to quantify muscle mass in elderly and young people and patients with CKD^12^^,^ 13. However, investigating the association between quadriceps muscle thickness by US and physical function in non-dialytic CKD patients will provide more information about how muscle impairment impacts functional performance in patients at the conservative treatment. Thus, the present study aimed to evaluate the relationship between quadriceps muscle thickness and functional performance on the 60s sit-to-stand test (60s-STS), six-minute walk test (6MWT), and handgrip strength in CKD patients non-dialytic stage.

## Methods

### Study design and setting

This was a cross-sectional study conducted from April 2018 to June 2019, where CKD patients from the Nephrology Service of the Clinical Hospital of the Universidade Federal de Pernambuco were recruited by convenience. All procedures were carried out at the Cardiopulmonary Physical Therapy Laboratory of the Universidade Federal de Pernambuco in Recife-PE, Brazil. This study was approved by the ethics and research committee of this institution (CAAE: 84135518.3.0000.5208 and opinion number: 3,366,668) and followed the norms of the Strengthening the Reporting of Observational Studies in Epidemiology (STROBE)^14^.

### Participants

Participants with CKD, non-dialytic, at stage 4 and 5 according to the criteria proposed by the Kidney Disease Improving Global Outcomes^15^, aged between 30 and 70 years and of both genders were included in the study. Patients who were on a dialysis program, who had been transplanted, and who were unable to perform the functional or clinical tests or answer the questionnaires were excluded.

### Outcome measures

Participants were submitted to an initial assessment that included sociodemographic data (age, gender), anthropometric measures (weight and height), self-reported number of comorbidities and time in months and cause of CKD. After, physical activity level, body composition quadriceps muscle thickness and functional performance tests were measured as described below.

#### Level of physical activity

The level of physical activity was assessed using the International Physical Activity Questionnaire (IPAQ - short version), validated for the Brazilian population. The questionnaire consisted of eight open questions that assessed time and frequency of performing walking and activities of moderate and vigorous intensity in the last week in order to evaluate the time spent per week in physical activities. For the purposes of this study we have categorized physical activity levels as follow: sedentary when participants reported frequency of activities being less than 5 days/week or less than 150 min/week and active, when participants were above those limits^16^.

#### Body composition

Body composition was assessed by electrical bioimpedance analysis (InBody R20^®^ - Dogok 2-Dong, Gangnam-gu, Seoul, Korea). The patient was previously instructed to eat at least two hours before the evaluation, wear light clothing, avoid physical activity, and empty their bladder prior to the evaluation. The patient remained in an orthostatic position during the test with their feet positioned over the electrodes on the digital scale surface and the other electrodes attached to a bar supported by the patient's hands. The bioimpedance lasted 30 seconds and was repeated in case of reading error^17^. Body weight (kg), body mass index (BMI, kg/m^2^), and appendicular skeletal muscle (ASM, kg) were obtained.

#### Quadriceps muscle thickness

Quadriceps muscle thickness was measured using a portable high-definition ultrasound (Sonoace R3^®^, Samsung Medison - South Korea) with the transducer positioned perpendicular on the anterior thigh. The assessment of muscle thickness was performed in the dominant segment, which was identified from a question about which leg the patient usually kicks with and performed with the patient in supine position, with a foam roller below the popliteal region of this limb. The transducer was positioned midway between the anterior superior iliac spine and the knee joint line, from which the cross-sectional image of the quadriceps muscle was obtained^18^^,^ 19. Three femoral quadriceps thickness (mm) measurements were performed from the frozen image of the muscle on the equipment monitor. For recording purposes, the average of the three values obtained was considered as long as the difference between them was less than 10%^18^^,^ 20.

### Functional performance tests

#### Lower limb resistance (60s-STS)

The 60s sit-to-stand test (60s-STS) was performed to evaluate lower limb resistance. The patient was instructed to stand and sit in a 46-cm-high chair without arm support and with their hands positioned on the contralateral shoulder for 60 seconds. Patients were instructed to complete as many repetitions as possible within the standardization of the correct test execution movement^21^^,^ 22.

#### Submaximal functional capacity

The six-minute walk test (6MWT) was performed according to the guidelines established by the American Thoracic Society^23^. The distance traveled was recorded in meters at the end of the test. The reference equation for the Brazilian population was used to estimate the predicted percentage^24^.

#### Handgrip strength

Handgrip strength was verified using a dynamometer (Dinamometer Smedley^®^ - hand type, Saehan - Korea) according to the guidelines established by The American Society of Hand Therapists^25^. The test was performed 3 times in the dominant limb, with a 30-second rest interval between each maneuver, and the highest value among the measurements was adopted provided that the difference between them was less than 10%^26^. The predicted value for each patient was determined from prediction equations proposed by the study by Novaes et al. (2009)^27^. Muscle strength was classified as low if values less than 32 kg for men and less than 17 kg for women were obtained, based on the 20^th^ percentile of the sample.

### Statistical analysis

The Kolmogorov-Smirnov test was initially used to verify the normality distribution of continuous variables. Data were expressed as mean and 95% confidence interval for continuous variables and as means of distribution of absolute and percentage frequency for dichotomous variables. Pearson's correlation test was used to assess the relationship between quadriceps thickness and appendicular skeletal muscle. Multiple linear regression was used to investigate the relationship between the quadriceps thickness and functional performance (60s-STS, 6MWT, and handgrip strength)^28^^,^ 29. Each functional performance variable was analyzed separately with quadriceps thickness in order to avoid multicollinearity. All models were adjusted for gender, age, and height. Data were processed using the Statistical Package for Social Science (SPSS) software, Chicago, IL, USA, version 20.0 for Windows. The tests were considered significant at the 5% level.

## Results

After screening for study participation, 192 of the 312 patients did not meet the inclusion criteria. Of the 120 eligible patients, 40 did not agree to participate because they lived in the country side of the state, 17 gave up due to financial difficulties, and 23 did not accept to commute to the evaluation location, thus a total of 40 patients were evaluated.

The general characteristics of the patients are described in Table 1. Mean age of the participants was 51.45 years, most participants were male (52.5%), sedentary (57.5%), and with disease time of 77.63 months.

**Table 1:** General characteristics of CKD study patients

Variables	Patients (n=40) Mean (95%CI) or n (%)
**Age** **Gender** Male	51.45 (48.26 - 54.64) 21 (52.5)
**CKD stage** 4 5	29 (72.5) 11 (27.5)
**PA level** Sedentary	23 (57.5)
**Comorbidities** SAH DM	36 (90) 14 (35)
**CKD time (months)**	77.63 (55.58 - 99.67)
**Body composition** Weight BMI (kg/m^2^) ASM (kg)	72.98 (68.40 - 77.56) 28.00 (26.50 - 29.50) 19.64 (18.28 - 20.99)

CKD: chronic kidney disease; PA: physical activity; SAH: systemic arterial hypertension; DM: diabetes mellitus; BMI: body mass index; ASM: appendicular skeletal muscle.

Table 2 shows the descriptive values of quadriceps thickness, functional performance tests and its predicted values. CKD patients walked 82.64% of the predicted 6MWT and 81.13% of them showed low values of handgrip strength.

**Table 2:** Quadriceps muscle thickness, 60s sit-to- stand test, distance covered in meters on the 6MWT and its predicted percentage, handgrip strength absolute values, and presence of muscle weakness in CKD patients

Variables	Patients (n=40)
	Mean (95%CI)/n (%)
**Quadriceps thickness (mm)**	23.92 (22.43 - 25.41)
**60s-STS**	22.93 (21.62 - 24.23)
**DT on 6MWT(m)**	471.23 (445.97 - 496.48)
**DT%**	82.64 (79.23 - 86.05)
**Handgrip strength (kg)**	33.27 (28.79 - 37.76)
**% Handgrip strength**	81.13 (73.42 - 88.84)
**Muscular weakness**	10 (25%)

60s-STS = 60s Chair sit-to-stand test; DT = distance traveled; 6MWT: six-minute walk test.

The quadriceps thickness was 26.60 (25.03-28.15) mm for men and 20.98 (19.01-22.94) mm for women. The performance on the 60s-STS was 24.81 (23.11-26.50) repetitions for men and 20.84 (19.19-22.50) repetitions for women. Regarding the 6MWT, 16 patients (40%) presented performance below 80% of the predicted value, with 28.6% men (6) and 52.6% women (10). The frequency of muscle weakness among men and women was 28.6% (6) and 21% (4), respectively.

According to the result of the multiple linear regression analysis, a relationship was observed between quadriceps thickness and number of repetitions on the 60s-STS, where the increase by one repetition was related to a 0.34 mm increase in quadriceps thickness (R^2^ = 0.436; 95% CI: 0.022-0.665; p=0.03), as shown in Table 3. Moreover, quadriceps thickness was moderately correlated with ASM (r = 0.603, p < 0.001).

**Table 3:** Relationship between quadriceps muscle thickness and functional performance

Functional performance	Quadriceps muscle thickness				
	**β**	**R^2^**	**IC95%**		**p-value**
60s-STS (no. of repetitions)	0.343	43.6%	0.022	0.665	0.037
Distance traveled (m)	-0.008	37.3%	-0.026	0.011	0.404
Handgrip strength (kg)	0.065	38.1%	-0.055	0.185	0.282

6MWT: Six-minute walk test; 60s-STS: 60s sit-to-stand test. Linear regression, p <0.05. All models were adjusted for gender, age, and height.

## Discussion

A relationship between quadriceps muscle thickness with 60s-STS was observed in the study patients. To date, the only study with similar results was that of Wilkinson et al. (2019)^30^, who found a negative relationship between the low echogenicity of the rectus femoris measured by ultrasound and the 60s-STS in CKD patients under conservative treatment; however, according to these authors, the cross-sectional area of this muscle was considered the best predictor of physical performance in these patients, relative to echogenicity, as found in the present study.

Our study found a positive relationship between quadriceps thickness and 60s-STS, similar to that found in studies by Mateos-Angulo et al. (2019)^31^, Lopez et al. (2017)^32^, and McIntyre et al. (2006)^33^. Mateos-Angulo et al. (2019)^31^ and Lopez et al. (2017)^32^ evaluated muscle thickness by ultrasound and functional tests (5R-CST and 30R-CST, respectively) in older adults. McIntyre et al. (2006)^33^ used CT to verify the quadriceps cross-sectional area, relating it to the 60s-STS and also found similar results when comparing CKD patients under conservative treatment with those undergoing dialysis therapy. However, Segura-Ortí et al. (2018)^34^ found no relationship between quadriceps cross-sectional area and 5-repetition sit-to-stand test using MRI in conservative, hemodialysis, and CKD subjects, but found a strong relationship between isokinetic and isometric strength of this muscle. Changes occur in physical and functional performance of patients with CKD and are due to muscle protein degradation^35^, which can be observed by imaging exams^31^^-^ 34. The association found in our study reinforces the importance of periodic measurement of both tests since US is a low cost, portable method, and has good reliability that can complement functional tests to monitor the repercussions of the disease on the musculoskeletal system.

Our study found no relationship between quadriceps thickness and 6MWT. Wilkinson et al. (2019)^30^ evaluated the cross-sectional area of rectus femoris muscle of CKD patients under conservative treatment by ultrasound and found a moderate association with the incremental shuttle walking test, which is opposite to our findings. In CKD patients, factors linked to muscle quality (infiltration of fat and collagen into the muscle of these patients) and muscle size could result in muscle impairment contributing for structural changes in contractile properties in these patients^30^ leading to muscle loss, inability to exercise, and immobility^36^.

The 6MWT assesses patients' health status and involves an integrative analysis among cardiovascular, pulmonary, neuromuscular, and metabolic systems^23^. Functional disability in patients with CKD is multifactorial^37^, associated with cardiovascular disease^38^^,^ 39, sedentary behavior^40^^,^ 41, muscle weakness, and reduced glomerular filtration rate^5^^,^ 42 ^,^ 43. In this way, quadriceps muscle thickness, which represents only the amount of muscle mass, might not be related to the distance covered in the 6MWT.

Our study also found no relationship between quadriceps thickness and handgrip strength. These results suggest that upper limb strength measurements are not comparable to measurements of quadriceps thickness, although both reflect on patient functionality. CKD patients have reduced strength and muscle mass, characterizing sarcopenia^6^^,^ 44, although we only observed 5% of sarcopenia in our sample (data not shown). According to the current European Consensus on Older Adult Sarcopenia^6^, muscle strength is the best predictor of adverse outcomes (falls, fractures, physical dysfunction, and mortality) for patients when compared to only reduction/loss in muscle mass, and its periodic evaluation in CKD is necessary.

A moderate correlation was found between muscle thickness and the ASM in our patients, which could explain the relationship between the thickness of the muscle and its functional performance. Changes in body composition are present in early stages of CKD as a consequence of uremic syndrome, and these lead to increased muscle proteolysis and reduced protein synthesis^5^^,^ 33 ^,^ 45. The reduction in muscle mass and muscle strength may cause impairment in physical and functional performance of these patients as GFR decreases^5^^,^ 34 ^,^ 35.

Muscle weakness was observed in 25% of our patients under conservative treatment. A relatively recent study found that muscle mass and strength reduction in dialysis patients were predictors of mortality in this population but muscle strength reduction was more strongly associated with mortality^44^. However, we highlight the fact that the patients evaluated in our study were under conservative treatment and had already presented muscle weakness. Considering that these patients will progress to the final stages of CKD, a marked reduction in muscle mass and strength is expected, which may result in decreased functional performance^35^.

Regarding functional performance on the 6MWT of our patients, we observed that 40% of them were below 80% of the predicted distance traveled. In evaluating patients on peritoneal dialysis and hemodialysis program, Painter et al^46^ identified 62.2 and 52.8% of the predicted 6MWT, respectively. Faria et al. (2013)^47^ observed a progressive decline in the distance covered on the 6MWT by patients with CKD as they advanced in disease staging. Segura-Ortí et al. (2018)^34^ also found a shorter distance traveled on this test between patients on conservative and dialytic treatment when compared to healthy individuals. The reduction in the functional capacity of our patients in the conservative stage calls for preventive measures to be instituted in order to prevent functional decline in later stages of the disease, when patients may progress to terminal CKD. For clinical implications, considering that almost 60% of our sample was sedentary, exercise programs for this population need to be implemented as a strategy to maintain physical activity level.

## Conclusion

In the present study, we found a correlation between quadriceps muscle thickness assessed by ultrasonography and functional performance on the 60s-STS and between this muscle thickness and ASM in CKD patients under non-dialytic treatment. As a future perspective, imaging evaluation and functional tests may be considered in clinical practice, as their results could be used for physical therapy monitoring to improve functional status in exercise programs developed to minimize the deleterious effects of CKD.

### Limitations of the study

The present study had as limitation the lack of echogenicity quadriceps assessment. Another possible limitation was that we did not include patients at all CKD stages, which could provide additional information about which stage of the disease occurs the greatest impairment in physical function. Finally, we have not performed a prior sample size calculation. However, a post-hoc power analysis using four predictors and the probability level of 0.05, resulted in a power of 0.99, 0.97, and 0.97, respectively^48^.
